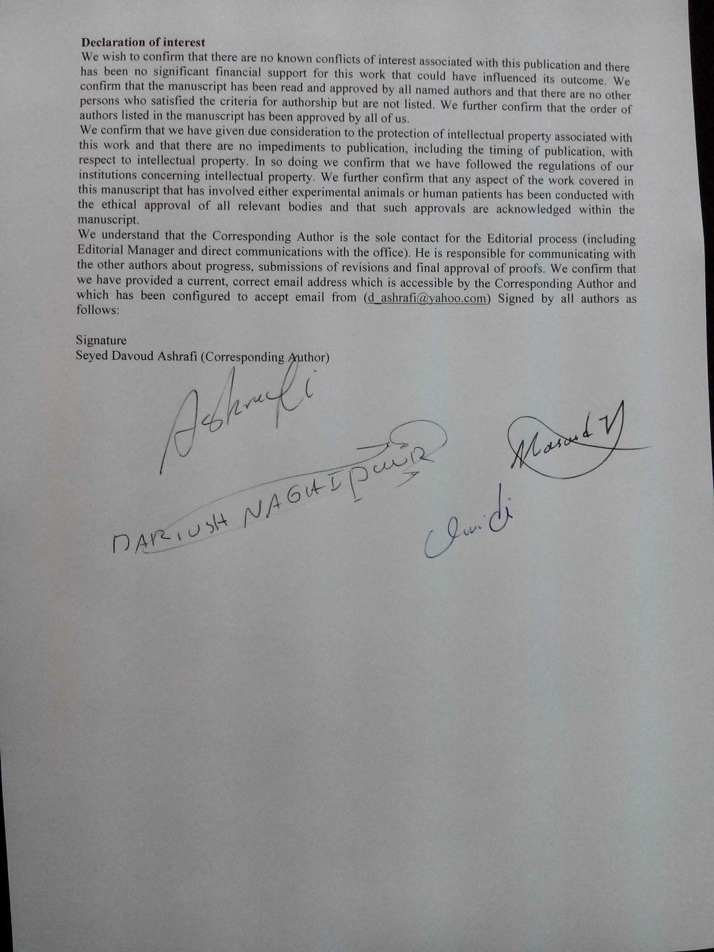# Survey and mapping of heavy metals in groundwater resources around the region of the Anzali International Wetland; a dataset

**DOI:** 10.1016/j.dib.2018.03.058

**Published:** 2018-03-19

**Authors:** Masoud Vatandoost, Dariush Naghipour, Saeed Omidi, Seyed Davoud Ashrafi

**Affiliations:** a School of Health, Guilan University of Medical Sciences, Rasht, Iran; b Research Center of Health and Environment, Guilan University of Medical Sciences, Rasht, Iran

**Keywords:** Heavy metal, Groundwater, Anzali wetland, GIS

## Abstract

The purpose of this study is zoning and determining the concentration of heavy metals including Arsenic (As), Mercury (Hg), Lead (Pb), and Cadmium (Cd) in the groundwater resources of villages located around the Anzali International Wetland. The amount of heavy metals (As, Hg, Pb, and Cd) in the collected samples were determined by the Inductively Coupled Plasma Optical Emission Spectrometry (ICP-OES) technique. The maximum concentrations of As, Hg, Pb and Cd were 0.216, 0.059, 0.090 and 0.006 mg/L, respectively.

**Specifications table**

**Table:** 

**Subject area**	Environmental Sciences
**More specific subject area**	Drinking water monitoring
**Type of data**	Table and figure
**How data was acquired**	Measurements of all parameters was done according to standard methods [1];
	pH was analyzed by digital pH meter (Metrohm).
	Digital thermometer was applied for temperature determination.
	Heavy metals were measured using Inductively Coupled Plasma (ICP-OES) technique.
	Scaling method was used for Total Dissolved Solids (TDS) analyzing.
	EC of water was measured by electrical conductivity meter.
**Data format**	Raw, analyzed
**Experimental factors**	The data were obtained in both dry and wet season, summer and winter, and the Electrical Conductivity (EC), pH and temperature were measured in the place, and the other samples were transferred to the laboratory for TDS and heavy metals measurements.
**Experimental features**	All studied parameters were determined and compared with standards [2], [3]. A spatial distribution map of heavy metals and weighted interpolation was made using the Arc GIS.
**Data source location**	Guilan Province, Iran (Fig. 1).
**Data accessibility**	All data are available.

**Value of the data**

• The data will be useful for health risk assessment of heavy metals related drinking water consumption.
• The data shown here can be helpful for Ministry of Power, water and wastewater companies for managing of groundwater resources.
• The zoning of the heavy metals was done to make a clear picture of the heavy metals concentrations in the groundwater resources of studied area.

## 1 Data

The contamination of groundwater is one of the most important environmental issues in the world [4], [5], [6], [7], [8], [9]. Among the various pollutants that affect water resources, pollutants containing heavy metals are particularly important due to their high toxicity, even at low concentrations [10], [11], [12], [13]. The parameters in the experiments of this research are including pH, TDS, EC, temperature and heavy metals (As, Hg, Pb, and Cd), in both season of winter and summer. The mean and standard deviation of the heavy metals concentrations and the physico-chemical parameters including pH, temperature, TDS and EC for both wet and dry seasons were given in Table 1. The statistical description of the concentration of heavy metals of water samples in the two seasons was given in Table 2. The average concentration of heavy metals in two seasons, for all studied parts and regions were given in Table 3. The Comparison of the average concentration of heavy metals in the dry and wet seasons was shown in Fig. 2, and the results of zoning the average concentrations of heavy metals evaluated in the groundwater of the study area in both the dry and wet seasons were shown in Fig. 3, Fig. 4. The data of statistical comparison of the average concentration of heavy metals in the dry and wet seasons were given in Table 4. The results of statistical tests (one-way Analysis of variance, ANOVA) to compare the average concentration of heavy metals in two seasons in the eastern, central, and western parts of the study area were given in Table 5.

**Fig. 1:**
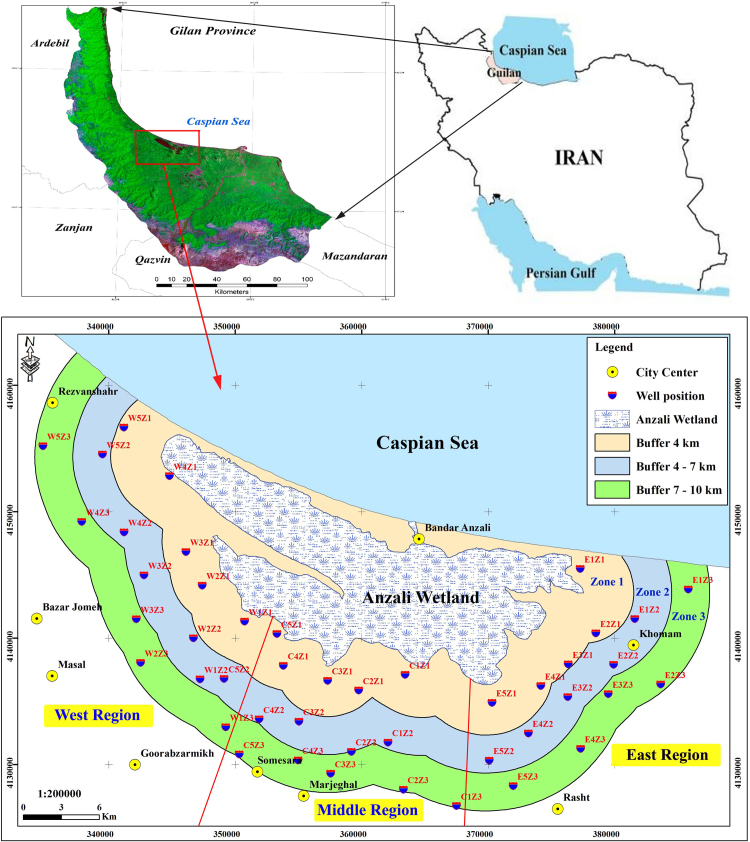
Geographical position of the triple studied area and sampling point.

**Fig. 2:**
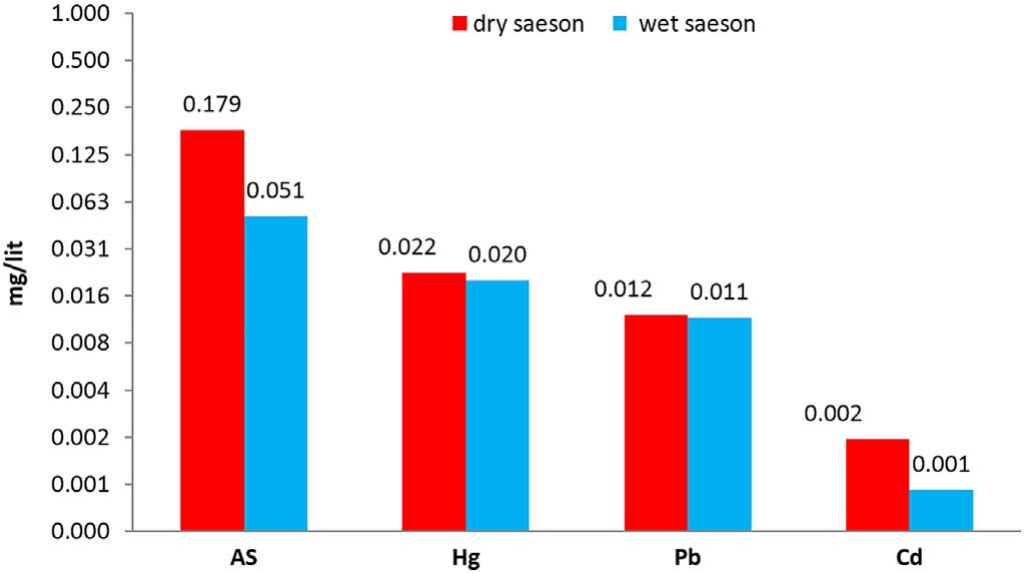
Average values of heavy metals in the study area in wet and dry season.

**Fig. 3:**
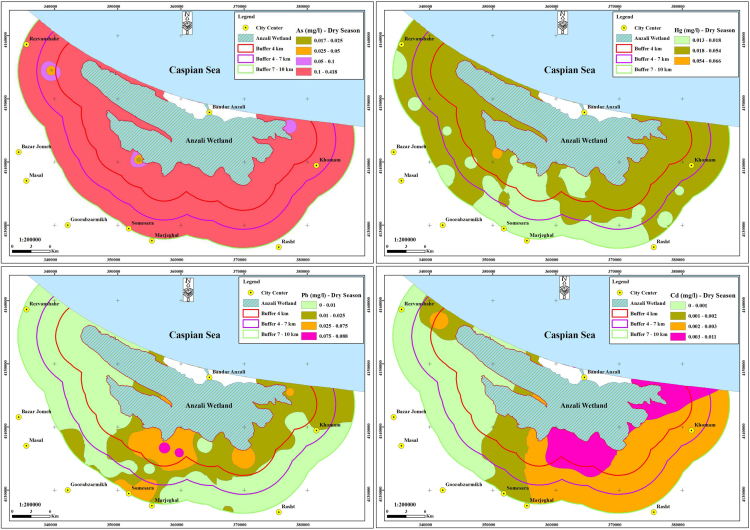
Zoning map of Arsenic, mercury, lead and cadmium concentrations in studied area in the dry season.

**Fig. 4:**
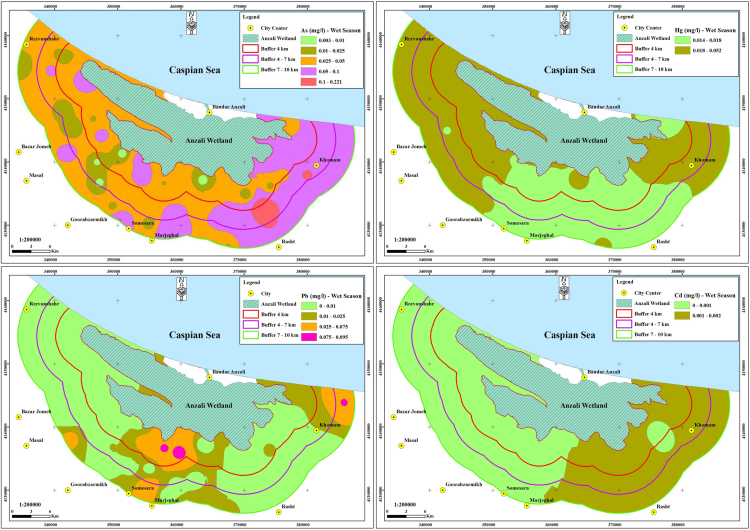
Zoning map of Arsenic, mercury, lead and cadmium concentrations in studied area in the wet season.

**Table 1:** The standards, mean and standard deviation values of parameters in both of wet and dry seasons.

**Parameter**	**Unit**	**Dry season**		**Wet season**		**Maximum permissible**	
		**Mean**	**Std. D**	**Mean**	**Std. D**	**National standard**	**WHO guideline**
**As**	mg/l	0.179	0.062	0.051	0.040	0.01	0.01
**Hg**	mg/l	0.022	0.011	0.020	0.007	0.006	0.006
**Pb**	mg/l	0.012	0.022	0.011	0.023	0.01	0.01
**Cd**	mg/l	0.002	0.002	0.001	0.001	0.003	0.003
**EC**	µS/cm	741.156	256.530	718.780	254.940	–	–
**TDS**	mg/l	370.578	128.260	359.390	127.470	1500	–
**pH**	–	7.781	0.221	7.652	0.310	6.5-9	–
**T**	°C	20.698	1.775	19.271	1.944	–	–

**Table 2.** The statistical description of the concentration of heavy metals.

**Heavy metals**	**Unit**	**Min**	**Max**	**Mean**	**Std. D**
**As**	mg/l	0.039	0.216	0.115	0.036
**Hg**	mg/l	0.014	0.059	0.021	0.008
**Pb**	mg/l	0.000	0.090	0.012	0.022
**Cd**	mg/l	0.000	0.006	0.002	0.001

**Table 3:** The average concentration of heavy metals in two seasons, for all studied parts and regions.

**Heavy metals**	**Unit**	**Parts**			**Regions**		
		**East**	**Center**	**West**	**1**	**2**	**3**
**As**	mg/l	0.137	0.107	0.100	0.107	0.119	0.118
**Hg**	mg/l	0.021	0.016	0.025	0.027	0.019	0.017
**Pb**	mg/l	0.009	0.023	0.002	0.020	0.003	0.012
**Cd**	mg/l	0.002	0.002	0.001	0.002	0.001	0.001

**Table 4:** Comparison of mean concentration of heavy metals in wet and dry seasons.

**Season**	**Heavy metals**	**One-sample t- Test**		
		**t**	**df**	**p-value**
**Wet season**	As	6.846	44	0.000
	Hg	13.682	44	0.000
	Pb	0.395	44	0.690
	Cd	−23.500	44	0.000
**Dry season**	As	18.341	44	0.000
	Hg	10.041	44	0.000
	Pb	0.559	44	0.580
	Cd	−3.511	44	0.000

**Table 5:** Comparison of the mean concentration of heavy metals in the eastern, middle and western parts and in the three regions of the study area.

**Parameter**	**(I) parts**	**(J) parts**	**Std. D (I - J)**	**P-value**	**(I) region**	**(J) region**	**Std. D (I-J)**	**P-value**
**As**	East	Center	0.030	0.327	1	2	−0.012	0.854
		West	0.038	0.179		3	−0.011	0.870
	Center	East	−0.031	0.327	2	1	0.012	0.854
		West	0.008	0.933		3	0.001	0.999
	West	East	−0.038	0.179	3	1	0.011	0.870
		Center	−0.008	0.933		2	−0.001	0.999
**Hg**	East	Center	0.006^*^	0.040	1	2	0.008^*^	0.000
		West	−0.004	0.181		3	0.009^*^	0.000
	Center	East	−0.006^*^	0.040	2	1	−0.008^*^	0.000
		West	−0.009^*^	0.000		3	0.002	0.665
	West	East	0.004	0.181	3	1	−0.009^*^	0.000
		Center	0.009^*^	0.000		2	−0.002	0.665
**Pb**	East	Center	−0.014^*^	0.029	1	2	0.017^*^	0.007
		West	0.007	0.402		3	0.008	0.355
	Center	East	0.014^*^	0.029	2	1	−0.017^*^	0.007
		West	0.021^*^	0.001		3	−0.009	0.196
	West	East	−0.007	0.402	3	1	−0.007	0.355
		Center	−0.021^*^	0.001		2	0.009	0.196
**Cd**	East	Center	0.001	0.808	1	2	0.001	0.081
		West	0.002^*^	0.000		3	0.001	0.081
	Center	East	−0.001	0.808	2	1	−0.001	0.081
		West	0.001^*^	0.003		3	0.000	1.000
	West	East	−0.002^*^	0.000	3	1	−0.001	0.081
		Center	−0.001^*^	0.003		2	0.000	1.000

## 2 Experimental design, materials and methods

### 2.1 Study area description

The study area is a part of the lowland plains of Foumanat (northern section) in Guilan province and is located in north of Iran (Fig. 1). Foumanat lowland is a part of the Anzali wetland watershed and the Caspian Sea, with area of 3,828.8 square kilometers. Sampling point include public and private wells that are the main sources of drinking water for local people. The locations of wells were recorded using geological positioning system (GPS).

### 2.2 Sample collection and analytical procedures

The study area was partitioned into three radial areas and three geographical sections of east, center, and west areas (Fig. 1). Forty five active deep wells in these nine sections were selected by systematic random sampling and for each sheet, five wells were taken for sampling. The 90 samples were collected in summer of 2016 and in winter of 2017. The sample containers were washed three times with distilled water and from each well 1.5 liter of water sample was obtained. The parameters of temperature, EC, TDS and pH were measured in place by portable devices. The other samples were fixed by nitric acid and stored in a dark cold box (4 °C) and transferred to laboratory for analyzing of heavy metals. Statistical analysis of data was done using SPSS 22 and the spatial variability and estimation of the concentration of heavy metals (preparation of zoning map) in the study area, was done by the Inverse Distance Weighting (IDW) method with Arc GIS software, version 10.1.

## Figures and Tables

**Fig. S1:**